# Subcommissural Organ-Spondin-Derived Peptide Restores Memory in a Mouse Model of Alzheimer’s Disease

**DOI:** 10.3389/fnins.2021.651094

**Published:** 2021-06-14

**Authors:** Juliette Le Douce, Nathalie Delétage, Valérie Bourdès, Sighild Lemarchant, Yann Godfrin

**Affiliations:** ^1^Axoltis Pharma, Lyon, France; ^2^Godfrin Life-Sciences, Caluire-et-Cuire, France

**Keywords:** peptide, subcommissural organ-spondin, Alzheimer’s disease, memory, drug candidate

## Abstract

Alzheimer’s disease (AD) is a devastating neurodegenerative disease that affects millions of older people worldwide and is characterized by a progressive deterioration of cognitive functions, including learning and memory. There are currently very few approved treatments (i.e., acetylcholinesterase inhibitors such as donepezil), all of which are limited to the symptomatic control of AD and are associated with side effects that may result in discontinuation of treatment. Therefore, there is an urgent need to develop disease-modifying treatments to prevent AD-induced cognitive deficits. Subcommissural organ (SCO)-spondin is a brain-specific glycoprotein produced during embryogenesis and has a substantial impact on neuronal development. In the current study, we sought to evaluate the protective effects of the linear (NX210) and cyclized (NX210c) forms of a SCO-spondin-derived peptide on learning and memory in a mouse model of AD. Mice received an intracerebroventricular injection of Aβ_25__–__35_ oligomers and were subsequently treated with intraperitoneal injections of vehicle, NX210 or NX210c of different doses (ranging from 0.1 to 30 mg/kg) and therapy paradigms (early or late stand-alone treatments, combination with donepezil or second-line treatment). Cognitive function was evaluated using Y-Maze, step-through latency passive avoidance (STPA) and Morris water maze (MWM) tests for up to 4 months. Early stage daily treatment with NX210 and NX210c decreased the levels of common pathological markers and features of AD, including Aβ_1__–__42_, phosphorylated-tau, inflammation, astrogliosis and lipid peroxidation. Meanwhile, use of these drugs increased the levels of synaptophysin and postsynaptic density protein 95. Regardless of the experimental paradigm used, NX210 and NX210c prevented Aβ_25__–__35_-induced decrease in spontaneous alternations (Y-Maze) and step-through latency into the dark compartment (STPA), and Aβ_25__–__35_-induced increase in time needed to locate the immersed platform during the learning phase and decrease in time spent in the target quadrant during the retention phase (MWM). Interestingly, this study provides the novel evidence that the native and oxidized cyclic forms of the SCO-spondin-derived peptide reduce pathological factors associated with AD and restore learning and memory at both early and late disease stages. Overall, this study sheds light on the therapeutic potential of this innovative disease-modifying peptide to restore memory function in patients with AD.

## Introduction

An increase in the prevalence of chronic diseases, including Alzheimer’s disease (AD), has accompanied the increase in aging population worldwide. Nearly 50 million people suffer from dementia, for which AD represents the leading cause worldwide and is associated with a high rate of unmet medical needs. Additionally, the prevalence of this disease is projected to more than triple by 2050 (Prince et al., 2015; Patterson, 2018; GBD 2016 Dementia Collaborators, 2019). It is estimated that approximately 5.8 million people aged over 65 years are currently living with AD dementia in the United States of America (Longhe, 2020). AD is a complex, multifactorial, age-related neurodegenerative disease characterized by the deposition of amyloid-beta (Aβ) peptides, formation of intracellular tau tangles and activation of other deleterious pathways (such as excitotoxicity, inflammation, oxidative stress, and synapse and neurovascular dysfunctions), which impede neuronal function and, ultimately, lead to neuronal death and cognitive decline (Heneka et al., 2015; Bejanin et al., 2017; Kisler et al., 2017; Tönnies and Trushina, 2017; Wang and Reddy, 2017; Jackson et al., 2019; Breijyeh and Karaman, 2020; Ayton and Bush, 2021). The current standards of care for the treatment of AD include therapies targeting the disturbances of the cholinergic and glutamatergic systems using drugs, such as donepezil (Aricept^®^), a reversible acetylcholinesterase inhibitor, and memantine (Ebixa^®^), an uncompetitive N-methyl-D-aspartate (NMDA) receptor antagonist, or a combination of these agents (Namzaric^®^) (Ferreira-Vieira et al., 2016; Arvanitakis et al., 2019; Conway, 2020). Overall, available pharmacological treatments are effective in reducing symptoms but not curing them. Among these treatments, studies have supported the efficacy of donepezil in symptom reduction, which is considered the mainstay treatment for patients with mild to severe AD despite dose-dependent adverse effects and modest clinical efficacy in treating cognitive decline (Tsoi et al., 2016; Arvanitakis et al., 2019; Haake et al., 2020). Although numerous anti-AD agents (>100) are being developed, there are currently no approved disease-modifying treatments (Cummings et al., 2019). This represents a major limitation in the attempt to reduce the rate of progression of AD pathology, which is necessary to reduce functional and cognitive deficits that result from AD. Therefore, efforts have been made to develop effective therapeutics at preclinical and clinical stages; among these, almost 30 anti-amyloid and anti-tau agents are in phase 3 (Breijyeh and Karaman, 2020; Haake et al., 2020; Zhang et al., 2020).

Subcommissural organ (SCO)-spondin is a large glycoprotein found in the central nervous system (CNS) during embryogenesis (Gobron et al., 1996). SCO-spondin comprises many conserved domains, including low-density lipoprotein receptor type A and epidermal growth factor-like domains, as well as thrombospondin type 1 repeats (TSR1), which are critical for protein–protein, cell–cell, and cell–extracellular matrix interactions that are necessary for neurogenesis and axonal pathfinding (Meiniel et al., 1996; Gobron et al., 1996; Vera et al., 2013, 2015; Stanic et al., 2014). NX210 is a native linear dodecapeptide derived from the most conserved sequence of the SCO-spondin TSR1 and has two cysteines that create a disulfide bond under oxidative conditions (e.g., in contact with blood or culture media), thereby forming a cyclic form of NX210, designated NX210c (Delétage et al., 2021). NX210 acts as a potent neuroprotective and neuroregenerative agent by preventing a decrease in neuronal viability induced by H_2_O_2_ and promoting neuritogenesis and neurite growth via integrins in the B104 neuronal cell line (Monnerie et al., 1998; Gobron et al., 2000; Bamdad et al., 2004; Sakka et al., 2014; Delétage et al., 2021). Local treatment of rats with spinal cord injury using NX210 resulted in increased axonal regrowth, collateral sprouting and, ultimately, functional recovery in aspiration and contusion models of spinal cord injury (Sakka et al., 2014). More recently, we have also shown a neuroprotective action of NX210c in glutamate-induced excitotoxicity in primary rat cortical and hippocampal neurons and human cortical neurons (Delétage et al., 2021). Furthermore, NX210c dose-response to trigger neuroprotection in rat neurons is more effective than that of NX210, suggesting that NX210c may represent the active form of the peptide (Delétage et al., 2021). Despite several reports on the neuroprotective and neuroregenerative properties of NX210 and NX210c, their therapeutic value as drug candidates for neurodegenerative diseases, including AD, remains unknown (Monnerie et al., 1998; Gobron et al., 2000; Bamdad et al., 2004; Sakka et al., 2014; Delétage et al., 2021).

In this study, we sought to evaluate the therapeutic effects of the SCO-spondin-derived NX210 peptide and its oxidized cyclic counterpart NX210c on learning and memory in a mouse model of AD induced by intracerebroventricular injection of Aβ_25__–__35_ oligomers (Maurice et al., 1996; Maurice et al., 2013; Chumakov et al., 2015; Droguerre et al., 2020). By using several biochemical analyses and behavioral tests in different therapy paradigms relevant to clinical conditions (early or late stand-alone treatments, combination with donepezil or second-line treatment), we provide the first evidence of the fact that systemic administration of NX210 or NX210c halts the progression of AD and restores memory function, which is maintained for up to 4 months, in a mouse model.

## Materials and Methods

### Animals

All experimental procedures were conducted in strict adherence to the European Union Directive of 22 September 2010 (2010/63/UE) and approved by the French Ministry of Research. A total of 318 Swiss male mice (Janvier, Saint Berthevin, France) were used in this study, and only three deaths were reported. During the experimental period, up to six mice were housed per cage in a controlled environment (temperature: 22 ± 2°C, humidity: 50 ± 10%, 12 h/12 h light-dark cycle with lights off at 7 pm) with free access to food and water. The group sizes used in this study were advised by the contract research organization which conducted the experiments (Amylgen) which has more than 12 years of expertise in the Aβ_25__–__35_ mouse model.

### AD-Like Mouse Model

Four- to six-week-old Swiss male mice were injected with Aβ_25__–__35_ oligomers, as described in previous literature (Maurice et al., 1996, 1998; Meunier et al., 2006; Villard et al., 2009, 2011). Briefly, Aβ_25__–__35_ and scrambled control peptides (Genepep, Saint-Jean-de-Védas, France) were solubilized in sterile bi-distilled water (3 mg/mL) and then incubated for 4 days at 37°C, to induce Aβ_25__–__35_ oligomerization (Maurice et al., 1996). Mice were anesthetized with isoflurane 2.5% for 5 min and 3 μL of solution containing 9 nmol of Aβ_25__–__35_ oligomers or scrambled control peptides were slowly administered into the right lateral ventricle using a 4-mm long, 28-gauge needle as described in the literature (Maurice et al., 1998).

### Treatment Groups

NX210 is a native linear dodecapeptide (sequence: H-WSGWSSCSRSCG-OH) derived from the consensus sequence of the thrombospondin type 1 repeat of SCO-spondin glycoprotein. NX210c is the cyclic form of NX210 whose two cysteines naturally create a disulfide bond under oxidative conditions. NX210 was synthesized by PolyPeptide (Strasbourg, France), while NX210c was manufactured and supplied by Genepep. The purity of NX210 and NX210c was assessed as 95–97 and 95–96%, respectively, by high-performance liquid chromatography. Each peptide form was solubilized at 10 mg/mL in sterile pyrogen-free water (Aguettant, Lyon, France), aliquoted for storage at –80°C, and thawed and diluted before use or used extemporaneously.

Donepezil was purchased from Sigma-Aldrich (Saint-Louis, MO, United States) and solubilized at 0.2 mg/mL in sterile pyrogen-free water, aliquoted for storage at 4°C, and then diluted and used extemporaneously.

Mice were randomly assigned to treatment groups. Vehicle (sterile pyrogen-free water), NX210, and NX210c were injected intraperitoneally once daily starting at 1 h after administration of Aβ_25__–__35_ oligomers or 11 days later for up to 4 months. Vehicle (sterile pyrogen-free water) was also administered intraperitoneally to control mice injected with scrambled control peptides. Both peptide forms were used at 0.1, 1, 2, 3.75, or 30 mg/kg. They were administered either alone or in combination with donepezil. Donepezil was administered orally by gavage using a cannula (Dominique Dutscher SAS, Bernolsheim, France) attached to a 1-mL syringe (Terumo, Tokyo, Japan) once daily starting 1 h after administration of Aβ_25__–__35_ oligomers for up to 4 months. It was used at 1 mg/kg (active dose) and at 0.25 mg/kg (sub-therapeutic dose; Droguerre et al., 2020) alone or in combination with sub-therapeutic doses of NX210 or NX210c (0.1 mg/kg).

Different cohorts of mice were used for this study:

– One cohort of mice was used to evaluate the effect of early stage treatment with several doses of NX210 or NX210c administered once daily alone and in combination with donepezil on short-term cognitive deficits using Y-maze (day 8, D8) and step-through passive avoidance (STPA; D10) tests. Key group mice from this cohort were sacrificed at D11 1 h after treatment injection to collect the hippocampi and prefrontal cortices for enzyme-linked immunosorbent assay (ELISA) and lipid peroxidation (LPO) analyses.
Another cohort of mice was used to evaluate the effect of early stage treatment with an active dose of NX210 (2 mg/kg), NX210c (2 mg/kg), or donepezil (1 mg/kg) administered once daily on short-term cognitive deficits using a Morris water maze (MWM; D9–D14) test.
– Another cohort of mice was used to evaluate the effect of late-stage treatment with NX210 or NX210c administered once daily from D11 until D38 on short-term cognitive deficits using Y-Maze (weekly from D8 until D36), MWM (D30–D35) and STPA (D38) tests.
– Another cohort of mice was used to evaluate the effect of early- and late-stage treatments with NX210c (stand-alone treatments, combination with donepezil or second-line treatment) on long-term cognitive deficits using a Y-Maze test (weekly from D8 until D120). For this experiment, mice were injected intracerebroventricularly with scrambled control peptides or Aβ_25__–__35_ oligomers and treated intraperitoneally once a day with (i) vehicle, (ii) NX210c (2 mg/kg) from D1 to D120, (iii) NX210c (2 mg/kg) from D11 to D38, (iv) NX210c (0.1 mg/kg) along with donepezil (0.25 mg/kg) administered orally from D1 to D120, or (v) successively with donepezil (1 mg/kg, D1–D43) and NX210c (2 mg/kg, D44–D78; 4 mg/kg, D79–D99; 8 mg/kg, D100–D113).

For the group of mice that performed a behavioral test on the same day that a treatment was planned, the latter occurred 1 h before the test.

### Behavioral Tests

Several aspects of cognitive function were assessed using behavioral tests. The mice were habituated to the rooms where the behavior was conducted 30 min before the tests. Halogens directed toward the walls were used to light the rooms where STPA (25 lux) and Y-Maze (50 lux) tests were conducted. The tests were performed during the light phase of the mouse day cycle by observers blinded to the treatment groups and quantified by different observers also blinded to the treatment groups.

#### Y-Maze

Spatial working memory was evaluated using a Y-Maze test, as described in the literature (Droguerre et al., 2020). The test set-up consists of three arms (40 cm × 3 cm × 13 cm) with gray polyvinylchloride walls. Mice were placed at the end of one arm, and successive arm entries were recorded for up to 8 min by two experimenters. The results are expressed as a percentage of spontaneous alternation as follows: (consecutive entries in all arms/maximum alternations) × 100, where the maximum alternations represent the total number of arm entries minus two.

#### Step-Through Passive Avoidance

Contextual long-term memory was evaluated using a STPA test, as described in the literature (Chumakov et al., 2015). The test set-up consists of one lighted compartment (15 cm × 20 cm × 15 cm) with white polyvinylchloride walls, one dark compartment with black polyvinylchloride walls and a grid floor delivering electrical shocks (Lafayette Instruments, Lafayette, LA, United States), and a guillotine door in between the two compartments. Mice were placed in the white compartment and the door was raised 5 s later. Once the mice placed all four paws on the grid floor of the dark compartment, the door was closed, and a 0.3-mA foot shock was delivered for 3 s. Twenty-four hours after the training session, latency to enter the dark compartment was measured for up to 300 s.

#### Morris Water Maze

Spatial learning and memory were evaluated using a MWM test, as described in the literature (Droguerre et al., 2020). Mice were placed in a circular pool (140-cm diameter and 40-cm height) filled with opaque water under reproducible environmental conditions (external cues and light intensity – 36 lux at the surface of the pool). To assess spatial learning, the latency to find an immersed platform was measured from three independent swims for five consecutive days using Viewpoint motion capture software (Viewpoint, Champagne-au-Mont-d’Or, France). The location of the platform was changed on each training day. Twenty-four hours after the last training session, a probe test was performed to evaluate spatial memory retention, in which mice were placed in the pool without the platform, and the time spent in each of the four quadrants was measured for 60 s using the Viewpoint software. The results are expressed as the percentage of time spent in the target quadrant (quadrant containing the platform for the last training) compared with the mean time spent in the other three quadrants.

### Biochemical Analyses

**FIGURE 1:**
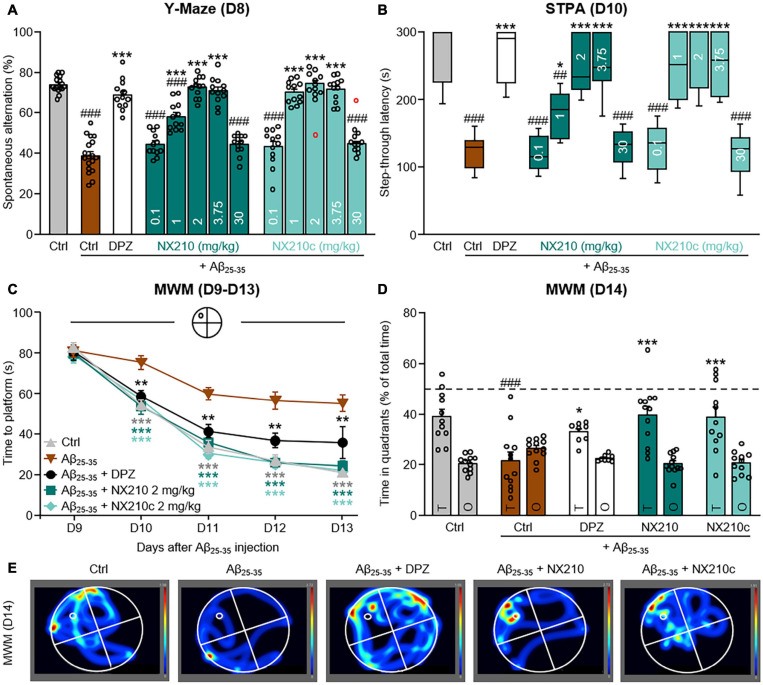
Early stage daily treatment with NX210 or NX210c prevents amyloid-beta_25__–__35_ (Aβ_25__–__35_)-induced short-term cognitive impairments in mice. Mice were treated intraperitoneally once a day with vehicle, NX210 or NX210c (0.1, 1, 2, 3.75, 30 mg/kg), or orally with donepezil (DPZ, 1 mg/kg) one hour after intracerebroventricular injection of scrambled control peptides (Ctrl) or Aβ_25__–__35_ oligomers. They were subjected to several behavioral tests to evaluate learning and memory. **(A)** Early stage daily treatment with NX210 and NX210c restored working memory deficit in a dose-dependent manner, as shown by the increased number of spontaneous alternations in the Y-maze assessed at day 8 (D8) compared with Aβ_25__–__35_-injected mice treated with vehicle. The data are presented as means and SEM. One-way ANOVA followed by Tukey’s multiple comparisons test: ^###^*p* < 0.001 compared with Ctrl; ***p < 0.001 compared with Aβ_25__–__35_, *n* = 16 Ctrl, *n* = 18 Aβ_25__–__35_, *n* = 11–12 Aβ_25__–__35_ + DPZ, Aβ_25__–__35_ + NX210, Aβ_25__–__35_ + NX210c. **(B)** Early stage daily treatment with NX210 and NX210c restored contextual long-term memory deficit in a dose-dependent manner, as shown by the increased time to enter the dark compartment in the step-through passive avoidance test performed at D10 compared with Aβ_25__–__35_-injected mice treated with vehicle. The data are presented as medians and interquartile range. Kruskal–Wallis followed by Dunn’s test: ^###^*p* < 0.001, ^##^*p* < 0.01 compared with Ctrl; ***p < 0.001, *p < 0.05 compared with Aβ_25__–__35_, *n* = 16 Ctrl, *n* = 18 Aβ_25__–__35_, *n* = 11–12 Aβ_25__–__35_ + DPZ, Aβ_25__–__35_ + NX210, Aβ_25__–__35_ + NX210c. **(A,B)** These analyses do not include two outliers identified for the Y-Maze using the Grubbs test (red circles) (*p* < 0.05). **(C–E)** Morris water maze test was performed from D9 to D14 to assess spatial learning **(C)** and retention memory **(D,E)** of mice treated with vehicle, NX210 or NX210c (2 mg/kg), or DPZ. Early stage daily treatment with NX210 and NX210c restored Aβ_25__–__35_-induced spatial learning and memory deficits, as shown by the decreased time to find the platform during the learning phase and the increased time spent in the target quadrant (T) versus other quadrants (O) during the retention phase in the Morris water maze compared with Aβ_25__–__35_-injected mice treated with vehicle. The data are presented as means and SEM. Two-way ANOVA followed by Tukey’s multiple comparisons test: **(C)** ***p < 0.001, **p < 0.01 compared with Aβ_25__–__35_; **(D)** ^###^*p* < 0.001 compared with Ctrl; ***p < 0.001, *p < 0.05 compared with Aβ_25__–__35_, *n* = 11 Ctrl, Aβ_25__–__35_ + NX210c, *n* = 12 Aβ_25__–__35_, Aβ_25__–__35_ + NX210, *n* = 8 Aβ_25__–__35_ + DPZ.

Control and Aβ_25__–__35_-injected mice treated with vehicle, NX210 or NX210c (2 mg/kg), or donepezil (1 mg/kg) that performed Y-Maze and STPA tests (Figure 1) were sacrificed by decapitation 10 days after injection of scrambled control peptides or Aβ_25__–__35_ oligomers (5–6 mice per group). The hippocampi and prefrontal cortices were quickly collected, rinsed in ice-cold phosphate-buffered saline, weighed, frozen in dry ice, and stored at –80°C until further processing was performed.

#### Enzyme-Linked Immunosorbent Assay

The left hippocampus and prefrontal cortex (both sides) samples were homogenized in a buffer containing 50 mM Tris and 150 mM NaCl (pH 7.5), sonicated for 20 s, and centrifuged at 16,100 × *g* for 15 min at 4°C to extract protein lysates. Based on the manufacturers’ instructions, ELISA for mouse amyloid-beta_1__–__42_ (Aβ_1__–__42_; Cloud-Clone Corp., Houston, TX, United States), phosphorylated-tau on threonine 181 (pTau; Thermo Fisher, Waltham, MA, United States), tumor necrosis factor alpha (TNF-α; Thermo Fisher) (from left hippocampi), glial fibrillary acidic protein (GFAP; Cloud-Clone Corp.), caspase-12 (Cloud-Clone Corp.) (from left prefrontal cortices), synaptophysin (Cloud-Clone Corp.) and postsynaptic density protein 95 (PSD95; Cloud-Clone Corp.) (from right prefrontal cortices) were performed. All samples were tested in duplicates, and the absorbance was read at 450 nm. The results are expressed in pg per mg of tissue.

#### Lipid Peroxidation

The right hippocampus samples were homogenized in cold methanol (1/10, w/v) and centrifuged at 1,000 × *g* for 5 min at room temperature (RT). Supernatants were incubated with a solution containing 1 mM FeSO_4_, 0.25 M H_2_SO_4_, and 1 mM xylenol orange for 30 min at RT, and absorbance was measured at 580 nm (A580_1_). Then, 10 μL of 1 mM cumene hydroperoxide (CHP) was added to the samples and incubated for 30 min at RT to determine the maximal oxidation level using absorbance at 580 nm (A580_2_). LPO levels were measured as follows: A580_1_/A580_2_ × CHP (nmol). The results are expressed as CHP equivalents per wet weight of tissue.

### Statistical Analyses

Few significant outliers were identified using a Grubbs test (*p* < 0.05; GraphPad QuickCalcs) for either behavioral tests or biochemical analyses and, therefore, were removed from all corresponding analyses. Statistical analyses were performed with GraphPad Prism software package 9.0.0 (GraphPad Software, La Jolla, CA, United States) using fixed-effect one-way or two-way analysis of variance (ANOVA) followed by Tukey’s multiple comparisons test for graphs containing groups that all passed a Shapiro–Wilk normality test and Brown–Forsythe test for group variances; otherwise, a non-parametric alternative to ANOVA was applied (i.e., Kruskal–Wallis followed by Dunn’s multiple comparisons test). A *p*-value < 0.05 was considered significant in all statistical tests. The data are expressed as mean ± standard error of the mean (SEM) values for the Y-Maze and MWM tests and biochemical analyses; whereas they are represented by a box depicting the median and the 25th and 75th quartiles, with whiskers showing the 5th and 95th percentiles for the STPA test.

## Results

### Early Stage Daily Treatment With NX210 or NX210c Prevents Short-Term Cognitive Decline in Aβ_25__–__35_-Injected Mice

Mice were treated daily with intraperitoneal injections of each peptide form at 0.1, 1, 2, 3.75, or 30 mg/kg starting 1 h after intracerebroventricular administration of Aβ_25__–__35_ oligomers to determine the effect of NX210 and NX210c on cognitive decline. Donepezil administration is one of the main standard of care treatments for AD due to its effect of increasing cholinergic neurotransmission; it was used as a positive control and administered orally at 1 mg/kg once a day starting 1 h after injection of Aβ_25__–__35_ oligomers. Spatial working memory, contextual long-term memory and spatial learning and memory were evaluated at 8, 10, and 9–14 days after injection of Aβ_25__–__35_ oligomers using the Y-Maze, STPA, and MWM tests, respectively (Figure 1).

Aβ_25__–__35_ oligomers significantly altered spatial working memory, as shown by a twofold reduction of spontaneous alternations during the Y-Maze test for AD-like mice compared with that for control mice (Figure 1A: *F*_12_,_151_ = 66.52, *p* < 0.0001. 74 and 38.9% of alternations in control and Aβ_25__–__35_-injected mice, respectively; *p* < 0.0001). Interestingly, both forms of the peptide restored working memory deficit in a dose-dependent manner. Indeed, 2 and 3.75 mg/kg doses fully prevented changes in Aβ_25__–__35_-induced spontaneous alternation (Figure 1A: 70.9–74.6% of alternations in Aβ_25__–__35_-injected mice treated with NX210 and NX210c; *p* < 0.0001 and >0.05 compared with Aβ_25__–__35_-injected treated with vehicle and control mice, respectively). Although NX210c at 1 mg/mL was also effective in preventing working memory decline in Aβ_25__–__35_-injected mice, this dose was only partially effective with NX210 (Figure 1A: 58.2 and 70.1% of alternations in Aβ_25__–__35_-injected mice treated with NX210 and NX210c, respectively; *p* < 0.0001 compared with Aβ_25__–__35_-injected mice treated with vehicle, *p* = 0.0006 between Aβ_25__–__35_-injected mice treated with NX210 and NX210c). However, no effects of NX210 and NX210c were observed at the lowest and highest doses used (Figure 1A: *p* = 0.4573, 0.5043, 0.8157, and 0.4574 for Aβ_25__–__35_-injected mice treated with NX210 and NX210c at 0.1 and 30 mg/kg doses, respectively). Overall, the most effective doses of NX210 and NX210c were as efficient as donepezil in restoring working memory (Figure 1A: 68.9% of alternations in Aβ_25__–__35_-injected mice treated with donepezil; *p* < 0.0001 and >0.05 compared with Aβ_25__–__35_-injected mice treated with vehicle and control mice, respectively).

Contextual long-term memory was also significantly reduced due to administration of Aβ_25__–__35_ oligomers, as shown by a twofold decrease in the step-through latency (STL) to the dark electrized compartment 24 h of the Aβ_25__–__35_-injected mice after being exposed to the electrical shock, compared with that of the control mice (Figure 1B: *H* = 124.6, *p* < 0.0001. 266.6 and 121 s for control and Aβ_25__–__35_-injected mice, respectively; *p* < 0.0001). NX210 and NX210c restored contextual long-term memory decline in a dose-dependent manner, similar to the effect observed in the results of the Y-Maze test. Indeed, 2 and 3.75 mg/kg doses prevented Aβ_25__–__35_-induced STL decrease (Figure 1B: 248.3–264.8 s for Aβ_25__–__35_-injected mice treated with NX210 and NX210c; *p* < 0.0001 and >0.05 compared with Aβ_25__–__35_-injected mice treated with vehicle and control mice, respectively). Although NX210c at 1 mg/mL was also effective in preventing contextual long-term memory deficits in Aβ_25__–__35_-injected mice, this dose was only partially effective with NX210 (Figure 1B: 177.9 and 252.4 s for Aβ_25__–__35_-injected mice treated with NX210 and NX210c, respectively; *p* = 0.0304 and < 0.0001 compared with Aβ_25__–__35_-injected mice treated with vehicle, *p* = 0.0164 between Aβ_25__–__35_-injected mice treated with NX210 and NX210c). However, no effects of NX210 and NX210c were observed at the lowest and highest doses used (Figure 1B: *p* = 0.9956, 0.6892, 0.6805, and 0.9909 for Aβ_25__–__35_-injected mice treated with NX210 and NX210c at 0.1 and 30 mg/kg doses, respectively). Overall, the most effective doses of NX210 and NX210c were as effective as donepezil in restoring contextual memory (Figure 1B: 265.8 s for Aβ_25__–__35_-injected mice treated with donepezil; *p* < 0.0001 and >0.05 compared with Aβ_25__–__35_-injected mice treated with vehicle and control mice, respectively).

In addition to spatial working memory and contextual long-term learning, NX210 and NX210c (2 mg/kg) also restored Aβ_25__–__35_-induced spatial learning and memory deficits in another cohort of mice. The latency to find the immersed platform in the pool was significantly increased in Aβ_25__–__35_-injected mice compared with that in control mice over the four training days [Figure 1C: *F*_16_,_196_ (mouse group × time) = 3.642, *p* < 0.0001. 21.1 s and 55.1 s for control and Aβ_25__–__35_-injected mice at D13, respectively; *p* < 0.0001]. Interestingly, Aβ_25__–__35_-injected mice treated with NX210 and NX210c demonstrated the same level of learning capacity as that of control mice (Figure 1C: 24.3 s and 22 s for Aβ_25__–__35_-injected mice treated with NX210 and NX210c at D13, respectively; *p* < 0.0001 and > 0.05 compared with Aβ_25__–__35_-injected mice treated with vehicle and control mice, respectively). Although Aβ_25__–__35_-injected mice treated with donepezil were as efficient as Aβ_25__–__35_-injected mice treated with NX210 and NX210c at locating the platform over the first training days, a significantly increased latency was observed at D13 compared with that of control mice (Figure 1C: 35.8 s; *p* = 0.0014 and 0.0365 compared with Aβ_25__–__35_-injected mice treated with vehicle and control mice, respectively). Furthermore, a trend of better performance of Aβ_25__–__35_-injected mice treated with NX210c compared to that of Aβ_25__–__35_-injected mice treated with donepezil was observed during the last training day (Figure 1C: *p* = 0.059). The good performance of Aβ_25__–__35_-injected mice treated with NX210 and NX210c was also confirmed during the probe test conducted 24 h after the last training day (i.e., D14), in which the platform was removed, and the time spent in each quadrant was measured to evaluate retention memory (Figures 1D,E). The Aβ_25__–__35_-injected mice spent half less time than did control mice in the target quadrant [Figure 1D: *F*_4_,_49_ (mouse group × quadrant type) = 5.592, *p* = 0.0009. 39 and 21.6 s for control and Aβ_25__–__35_-injected mice, respectively; *p* < 0.0001]. NX210 and NX210c (Figure 1D: 39.6 and 38.7 s for Aβ_25__–__35_-injected mice treated with NX210 and NX210c, respectively; *p* < 0.0001 and >0.05 compared with Aβ_25__–__35_-injected mice treated with vehicle and control mice, respectively), as well as donepezil (Figure 1D: 32.9 s; *p* = 0.0282 and >0.05 compared with Aβ_25__–__35_-injected mice treated with vehicle and control mice, respectively), were associated with reduced Aβ_25__–__35_-induced memory retention deficits. Conversely, the average time spent in the three other quadrants was similar among the groups (Figure 1D: *p* > 0.05).

### Early Stage Daily Treatment With NX210 or NX210c Reduces Levels of Cerebral Pathological Markers in Aβ_25__–__35_-Injected Mice

**FIGURE 2:**
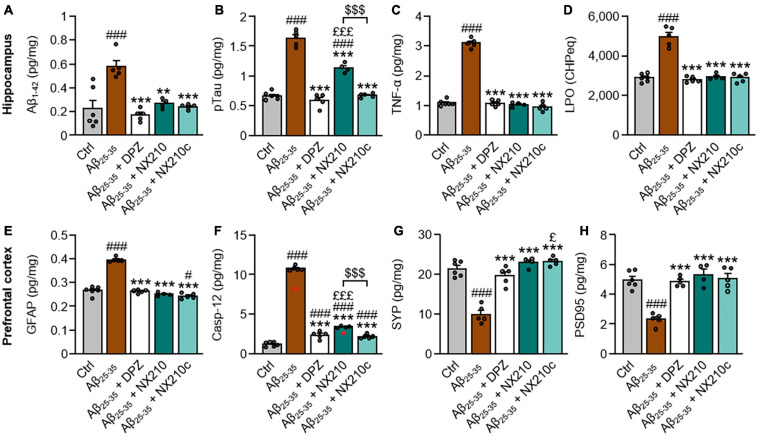
Early stage daily treatment with NX210 or NX210c reduces pathological hallmarks of Alzheimer’s disease. Mice were treated intraperitoneally once a day with vehicle, NX210 or NX210c (2 mg/kg), or orally with donepezil (DPZ, 1 mg/kg) one hour after intracerebroventricular injection of scrambled control peptides (Ctrl) or amyloid-beta_25__–__35_ (Aβ_25__–__35_) oligomers. They were sacrificed at day 11 (D11) and cerebral structures [hippocampi **(A–D)** and prefrontal cortices **(E–H)**] were collected for biochemical analyses of levels of Aβ_1__–__42_ (**A**; ELISA), phosphorylated-tau on threonine 181 [pTau; (**B**; ELISA)], tumor necrosis factor-α [TNFα; (**C**; ELISA)], lipid peroxidation [LPO; (**D;** measure of cumene hydroperoxide (CHP) absorbance)], glial fibrillary acidic protein [GFAP; (**E**; ELISA)], caspase-12 [Casp-12; (**F**; ELISA)], synaptophysin [SYP; (**G**; ELISA)] and postsynaptic density protein 95 [PSD95; (**H**; ELISA)]. NX210 and NX210c decreased the levels of all those pathological hallmarks of AD and increased synaptogenesis. The data are expressed in CHP equivalents (CHPeq) per wet weight of tissue **(D)** or in pg per mg of tissue (**other items**) and presented as means and SEM. One-way ANOVA followed by Tukey’s multiple comparisons test: ^###^*p* < 0.001, ^#^*p* < 0.05 compared with Ctrl; ***p < 0.001, **p < 0.01 comp ared with Aβ_25__–__35_; ^£££^*p* < 0.001, ^£^*p* < 0.05 compared with Aβ_25__–__35_ + DPZ; ^$$$^*p* < 0.001 Aβ_25__–__35_ + NX210 *vs.* Aβ_25__–__35_ + NX210c, *n* = 6 Ctrl, *n* = 5 Aβ_25__–__35_, Aβ_25__–__35_ + DPZ, Aβ_25__–__35_ + NX210c, *n* = 4 Aβ_25__–__35_ + NX210. These analyses do not include two outliers identified for caspase 12 ELISA using the Grubbs test (red circles) (*p* < 0.05).

We examined the protein levels of key pathological hallmarks of AD in the hippocampi and prefrontal cortices of control and Aβ_25__–__35_-injected mice treated or not with NX210 or NX210c (2 mg/kg), or donepezil (1 mg/kg) for 11 days, using ELISA for Aβ_1__–__42_, pTau, TNF-α, GFAP, caspase-12, synaptophysin and PSD95, and the measure of CHP absorbance for LPO. In this AD model, Aβ_25__–__35_ oligomer administration significantly increased Aβ_1__–__42_ and pTau levels, as well as markers of astrogliosis, inflammation, LPO, and endoplasmic reticulum stress; meanwhile, Aβ_25__–__35_ oligomers reduced the levels of synaptic markers synaptophysin and PSD95 (Figure 2: *p* ≤ 0.001 compared with control mice). Interestingly, NX210 and NX210c restored the basal levels of Aβ_1__–__42_ in the hippocampus of Aβ_25__–__35_-injected mice (Figure 2A: *F*_4_,_20_ = 12.84, *p* < 0.0001. + 0.3525, +0.046, and +0.0127 pg per mg of tissue in Aβ_25__–__35_-injected mice treated with vehicle, NX210 and NX210c, respectively, which corresponds to +155, +18, and +5.6% compared with that in control mice; *p* = 0.0015 and 0.0003 compared with Aβ_25__–__35_-injected mice treated with vehicle, *p* > 0.05 compared with control mice). Although Aβ_25__–__35_-injected mice treated with NX210c presented pTau levels similar to those of control mice, NX210 only partially reduced Aβ_25__–__35_-induced pTau increase in the hippocampus (Figure 2B: *F*_4_,_20_ = 123.4, *p* < 0.0001. + 0.9651, +0.4701, and +0.0071 pg per mg of tissue in Aβ_25__–__35_-injected mice treated with vehicle, NX210 and NX210c, respectively, which corresponds to +144.5, +70.4, and +1.1% compared with that in control mice; *p* < 0.0001 compared with Aβ_25__–__35_-injected mice treated with vehicle, *p* < 0.0001 and > 0.05 compared with control mice). NX210 and NX210c fully reversed the increases in TNF-α level (Figure 2C: *F*_4_,_20_ = 298.5, *p* < 0.0001. +2.006, –0.058, and –0.1425 pg per mg of tissue in Aβ_25__–__35_-injected mice treated with vehicle, NX210 and NX210c, respectively, which corresponds to +184.4, –5.3, and –13.1% compared with that in control mice; *p* < 0.0001 and >0.05 compared with Aβ_25__–__35_-injected mice treated with vehicle and control mice, respectively), LPO (Figure 2D: *F*_4_,_20_ = 58.24, *p* < 0.0001. + 2,077, +58, and +4 CHP equivalents per wet weight in Aβ_25__–__35_-injected mice treated with vehicle, NX210 and NX210c, respectively, which corresponds to +71.9, +2, and +0.1% compared with that in control mice; *p* < 0.0001 and >0.05 compared with Aβ_25__–__35_-injected mice treated with vehicle and control mice, respectively) and astrogliosis induced by Aβ_25__–__35_ (Figure 2E: *F*_4_,_20_ = 143.9, *p* < 0.0001. +0.1294, –0.0153, and –0.0233 pg of GFAP per mg of tissue in Aβ_25__–__35_-injected mice treated with vehicle, NX210 and NX210c, respectively, which corresponds to +48.7, –5.7, and –8.8% compared with that in control mice; *p* < 0.0001 and >0.05 compared with Aβ_25__–__35_-injected mice treated with vehicle and control mice, respectively). NX210 and NX210c partially prevented Aβ_25__–__35_-induced endoplasmic reticulum stress (Figure 2F: *F*_4_,_20_ = 840.4, *p* < 0.0001. +9.529, +2.191, and +0.964 pg of caspase-12 per mg of tissue in Aβ_25__–__35_-injected mice treated with vehicle, NX210 and NX210c, respectively, which corresponds to +787.1, +180.9, and +79.6% compared with that in control mice; *p* < 0.0001 compared with Aβ_25__–__35_-injected mice treated with vehicle, *p* < 0.0001 and = 0.0003 compared with control mice). Importantly, NX210 and NX210 protected Aβ_25__–__35_-injected mice from loss of presynaptic (Figure 2G: *F*_4_,_20_ = 44.48, *p* < 0.0001. –11.537, +1.53, and +1.83 pg of synaptophysin per mg of tissue in Aβ_25__–__35_-injected mice treated with vehicle, NX210 and NX210c, respectively, which corresponds to –53.8, +7.2, and +8.6% compared with that in control mice; *p* < 0.0001 and >0.05 compared with Aβ_25__–__35_-injected mice treated with vehicle and control mice, respectively) and postsynaptic markers (Figure 2H: *F*_4_,_20_ = 23.69, *p* < 0.0001. –2.654, +0.346, and 0.097 pg of PSD95 per mg of tissue in Aβ_25__–__35_-injected mice treated with vehicle, NX210 and NX210c, respectively, which corresponds to –53.6, +7, and +1.9% compared with that in control mice; *p* < 0.0001 and >0.05 compared with Aβ_25__–__35_-injected mice treated with vehicle and control mice, respectively). Overall, NX210c was found to be more effective than NX210 in restoring pTau and caspase-12 levels in Aβ_25__–__35_-injected mice (Figures 2B,F: *p* < 0.0001). Although donepezil also had a beneficial effect on protein levels of the aforementioned markers, the levels of synaptophysin were significantly higher in Aβ_25__–__35_-injected mice treated with NX210c than in those treated with donepezil (Figure 2G: +3.31 and +3.61 pg of synaptophysin per mg of tissue in Aβ_25__–__35_-injected mice treated with NX210 and NX210c compared with that in Aβ_25__–__35_-injected mice treated with donepezil, respectively, which corresponds to +15.5 and +16.9% compared with that in Aβ_25__–__35_-injected mice treated with donepezil; *p* = 0.0921 and 0.0395); however, protein levels were not different between Aβ_25__–__35_-injected mice treated with donepezil and control mice (Figure 2G: *p* = 0.5181).

### Early Stage Daily Combinatory Treatment With Donepezil/NX210 or Donepezil/NX210c at Sub-Therapeutic Doses Protects Aβ_25__–__35_-Injected Mice From Short-Term Cognitive Decline

**FIGURE 3:**
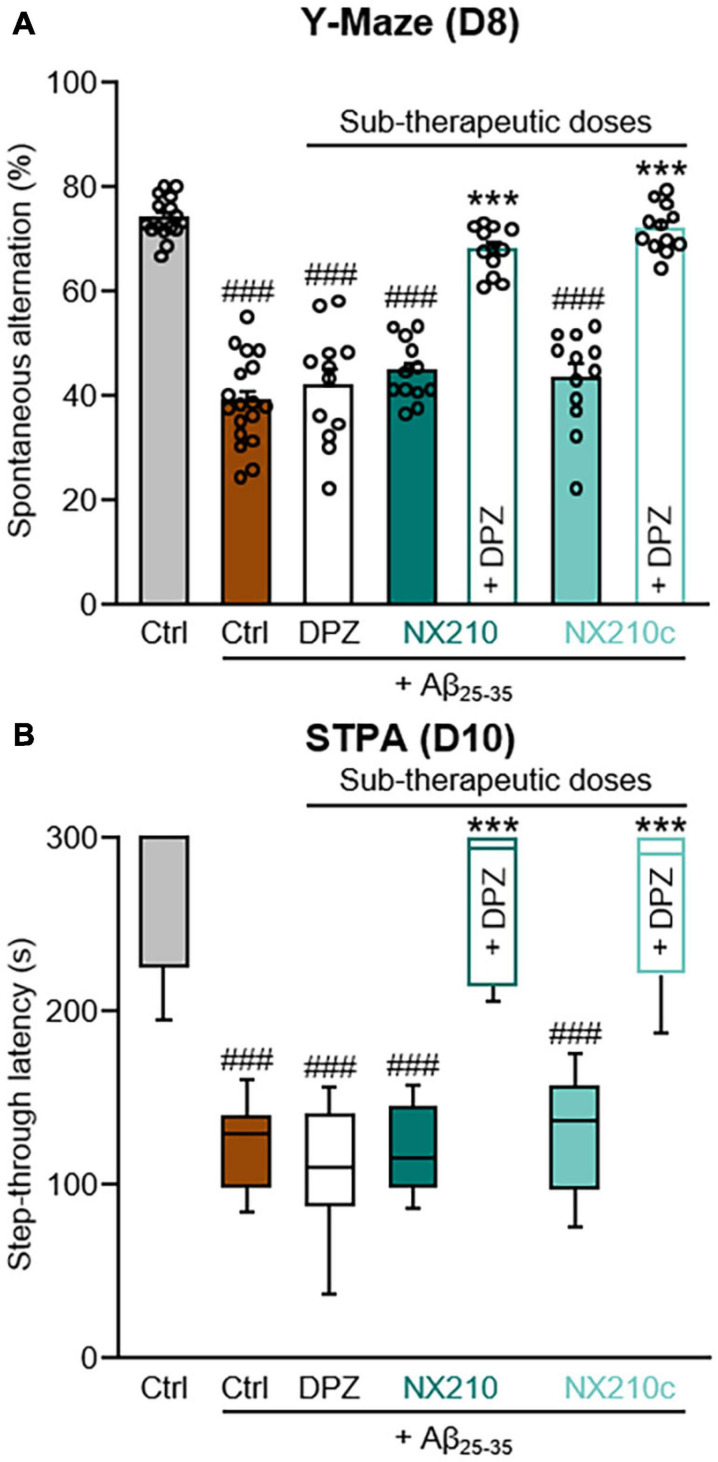
Early stage daily treatment with a combination of sub-therapeutic doses of donepezil and NX210 or donepezil and NX210c prevents amyloid-beta_25__–__35_ (Aβ_25__–__35_)-induced short-term cognitive impairments in mice. Mice were treated intraperitoneally once a day with vehicle, NX210 or NX210c (0.1 mg/kg), along with donepezil (DPZ, 0.25 mg/kg) administered orally one hour after intracerebroventricular injection of scrambled control peptides (Ctrl) or Aβ_25__–__35_ oligomers. They were subjected to several behavioral tests to evaluate learning and memory. **(A)** Early stage daily treatment with a combination of sub-therapeutic doses of donepezil and NX210 or donepezil and NX210c restored working memory deficit, as shown by the increased number of spontaneous alternations in the Y-maze assessed at day 8 (D8) compared with Aβ_25__–__35_-injected mice treated with vehicle. The data are presented as means and SEM. **(B)** Early stage daily treatment with a combination of sub-therapeutic doses of donepezil and NX210 or donepezil and NX210c restored contextual long-term memory deficit, as shown by the increased time to enter the dark compartment in the step-through passive avoidance test performed at D10 compared with Aβ_25__–__35_-injected mice treated with vehicle. The data are presented as medians and interquartile range. **(A,B)** Kruskal–Wallis followed by Dunn’s test: ^###^*p* < 0.001 compared with Ctrl; ***p < 0.001 compared with Aβ_25__–__35_, *n* = 16 Ctrl, *n* = 18 Aβ_25__–__35_, *n* = 12 Aβ_25__–__35_ + DPZ, Aβ_25__–__35_ + NX210, Aβ_25__–__35_ + NX210c, Aβ_25__–__35_ + (NX210 + DPZ), Aβ_25__–__35_ + (NX210c + DPZ).

Although donepezil represents a gold standard treatment for AD, it reportedly has dose-dependent side effects, notably on the gastrointestinal and urinary tracts (Arvanitakis et al., 2019; Haake et al., 2020). Therefore, we investigated the therapeutic effects of a daily combination treatment of donepezil and NX210 or NX210c at sub-therapeutic doses (0.25 and 0.1 mg/kg doses, respectively) starting 1 h after the administration of Aβ_25__–__35_ oligomers, as previously described (Figure 1), on spatial working memory and contextual long-term memory (Figure 3). Interestingly, donepezil/NX210 and donepezil/NX210c treatments substantially reduced the decline of spontaneous alternations observed in Aβ_25__–__35_-injected mice (Figure 3A: *H* = 71.18, *p* < 0.0001. 67.9 and 72% of alternations in Aβ_25__–__35_-injected mice treated with donepezil/NX210 and donepezil/NX210c; *p* < 0.0001 and >0.05 compared with Aβ_25__–__35_-injected mice treated with vehicle and control mice, respectively). In addition to improving spatial working memory, donepezil/NX210 and donepezil/NX210c treatments also prevented a decrease in STL to the dark electrized compartment, which was observed in Aβ_25__–__35_-injected mice treated with vehicle (Figure 3B: *H* = 69.82, *p* < 0.0001. 264.3 and 263.4 s for Aβ_25__–__35_-injected mice treated with donepezil/NX210 and donepezil/NX210c, respectively; *p* < 0.0001 and >0.05 compared with Aβ_25__–__35_-injected mice treated with vehicle and control mice, respectively).

### Late-Stage Daily Treatment With NX210 or NX210c Prevents Short-Term Cognitive Decline in Aβ_25__–__35_-Injected Mice

**FIGURE 4:**
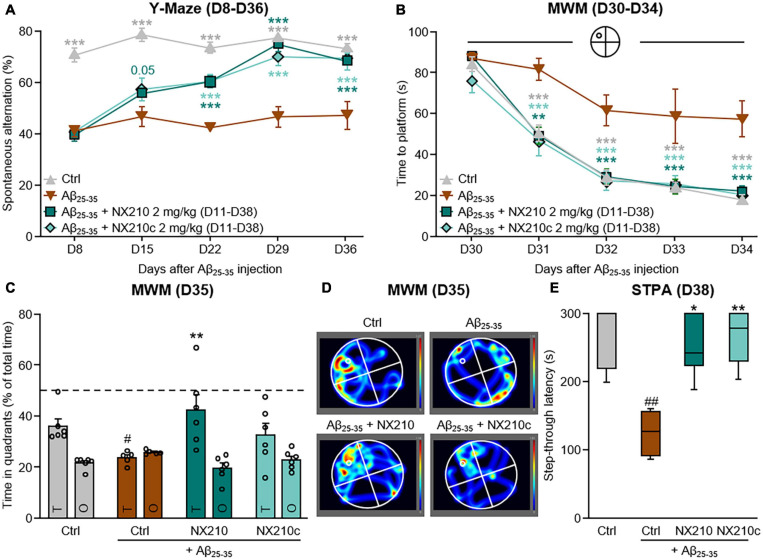
Late-stage daily treatment with NX210 or NX210c restores amyloid-beta_25__–__35_ (Aβ_25__–__35_)-induced short-term cognitive impairments in mice. Mice were injected intracerebroventricularly with scrambled control peptides (Ctrl) or Aβ_25__–__35_ oligomers, and treated intraperitoneally once a day with vehicle, NX210 or NX210c (2 mg/kg) from day 11 (D11) until the end of the experiment (D38). They were subjected to several behavioral tests to evaluate learning and memory. **(A)** Spontaneous alternation in the Y-maze was assessed weekly from D8 to D36 to study spatial working memory. Late-stage daily treatment with NX210 and NX210c restored working memory deficit, as shown by the increased number of spontaneous alternations in the Y-maze compared with Aβ_25__–__35_-injected mice treated with vehicle. The data are presented as means and SEM. Two-way ANOVA followed by Tukey’s multiple comparisons test: ***p < 0.001 compared with Aβ_25__–__35_. **(B–D)** Morris water maze test was performed from D30 to D35 to assess spatial learning **(B)** and retention memory **(C,D)**. Late-stage daily treatment with NX210 and NX210c restored Aβ_25__–__35_-induced spatial learning and memory deficits, as shown by the decreased time to find the platform during the learning phase and the increased time spent in the target quadrant (T) versus other quadrants (O) during the retention phase in the Morris water maze compared with Aβ_25__–__35_-injected mice treated with vehicle. The data are presented as means and SEM. Two-way ANOVA followed by Tukey’s multiple comparisons test: **(B)** ***p < 0.001, **p < 0.01 compared with Aβ_25__–__35_; **(C)** ^#^*p* < 0.05 compared with Ctrl; **p < 0.01 compared with Aβ_25__–__35_. **(E)** Late-stage daily treatment with NX210 and NX210c restored contextual long-term memory deficit in a dose-dependent manner, as shown by the increased time to enter the dark compartment in the step-through passive avoidance test performed at D38 compared with Aβ_25__–__35_-injected mice treated with vehicle. The data are presented as medians and interquartile range. Kruskal–Wallis followed by Dunn’s test: ^##^*p* < 0.01 compared with Ctrl; **p < 0.01, *p < 0.05 compared with Aβ_25__–__35_. **(A–D)** *n* = 6 Ctrl, Aβ_25__–__35_ + NX210, Aβ_25__–__35_ + NX210c, *n* = 5 Aβ_25__–__35_.

Mice were treated daily with each peptide form at 2 mg/kg starting 11 days after the administration of Aβ_25__–__35_ oligomers to determine the effect of NX210 and NX210c on cognitive decline after the disease was established (Figure 2). Spatial working memory, spatial learning and memory and contextual long-term memory were evaluated weekly from D8 to D36 (Y-Maze), from D30 to D35 (MWM), and at day 38 (STPA) following the injection of Aβ_25__–__35_ oligomers, respectively (Figure 4).

Long-term deficits in spatial working memory were observed for up to 36 days after administration of Aβ_25__–__35_ oligomers [Figure 4A: *F*_12_,_76_ (mouse group × time) = 4.848, *p* < 0.0001. 73.2 and 47% of alternations in control and Aβ_25__–__35_-injected mice, respectively; *p* < 0.0001]. Interestingly, late-stage daily treatments with NX210 and NX210c prevented changes in Aβ_25__–__35_-induced spontaneous alternation (Figure 4A: 68.2 and 69.5% of alternations in Aβ_25__–__35_-injected mice treated with NX210 and NX210c at D36, respectively; *p* < 0.0001 and >0.05 compared with Aβ_25__–__35_-injected mice treated with vehicle and control mice, respectively). Spatial learning and memory deficits were maintained over time. Indeed, the latency to find the immersed platform in the pool was significantly increased in Aβ_25__–__35_-injected mice compared with that in control mice over the four daily training days [Figure 4B: *F*_12_,_76_ (mouse group × time) = 2.125, *p* = 0.0246. 18 and 57.4 s for control and Aβ_25__–__35_-injected mice at D34, respectively; *p* < 0.0001]. Interestingly, Aβ_25__–__35__–_injected mice treated with NX210 and NX210c demonstrated the same learning capacity as that of control mice (Figure 4B: 22 and 20.3 s for Aβ_25__–__35_-injected mice treated with NX210 and NX210c at D34, respectively; *p* < 0.0001 and >0.05 compared with Aβ_25__–__35_-injected mice treated with vehicle and control mice, respectively). The good performance of Aβ_25__–__35_-injected mice treated with NX210 was also observed during the probe test at D35 (Figures 4C,D). Aβ_25__–__35_-injected mice spent less time in the target quadrant than did the control mice [Figure 4C: *F*_3_,_19_ (mouse group × quadrant type) = 3.258, *p* < 0.0001. 36 and 23.6 s for control and Aβ_25__–__35_-injected mice, respectively; *p* = 0.0465]. Although late-stage daily treatment with NX210 alleviated Aβ_25__–__35__–_induced retention memory deficits, no significant effect was observed with NX210c (Figure 4C: 42.3 and 32.3 s for Aβ_25__–__35_-injected mice treated with NX210 and NX210c, respectively; *p* = 0.0011 and 0.2375 compared with Aβ_25__–__35_-injected mice treated with vehicle, *p* > 0.05 compared with control mice). Conversely, the average time spent in the three other quadrants was similar among the groups (Figure 4C: *p* > 0.05). In addition to decline of spatial working memory and spatial learning and memory, contextual long-term memory was still impaired more than 5 weeks after Aβ_25__–__35_ injection as shown by a twofold decrease in the STL to the dark electrized compartment of Aβ_25__–__35_-injected mice, compared with that of control mice (Figure 4E: *H* = 12.43, *p* = 0.0060. 271 and 124.6 s for control and Aβ_25__–__35_-injected mice, respectively; *p* = 0.002). Late-stage daily treatment with NX210 and NX210c prevented Aβ_25__–__35_-induced STL decrease (Figure 4E: 251.2 and 266.7 s for Aβ_25__–__35_-injected mice treated with NX210 and NX210c, respectively; *p* = 0.0121 and 0.0023 compared with Aβ_25__–__35_-injected mice treated with vehicle, *p* > 0.05 compared with control mice).

### Early- and Late-Stage Daily Treatments With NX210c Prevent Long-Term Cognitive Decline in Aβ_25__–__35_-Injected Mice

In addition to having a slightly better efficacy/dose ratio than that of NX210 in this AD mouse model, NX210c stability and scale-up manufacturing are also more convenient than those of the linear form, which justifies future attempts to evaluate NX210c as a drug candidate rather than NX210. Therefore, we focused on the long-term benefits of NX210c in several therapy paradigms.

#### Early Stage Daily or Late-Stage Transient Daily Treatments With NX210c Prevent Long-Term Cognitive Decline in Aβ_25__–__35_-Injected Mice

**FIGURE 5:**
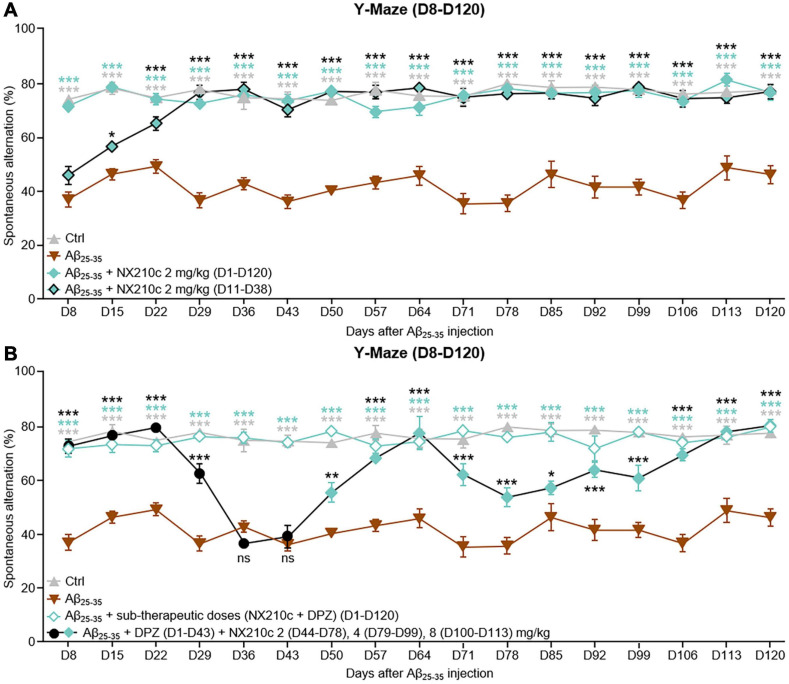
Early- and late-stage daily treatments with NX210c prevent amyloid-beta_25__–__35_ (Aβ_25__–__35_)-induced long-term cognitive impairments in mice. **(A)** Mice were injected intracerebroventricularly with scrambled control peptides (Ctrl) or Aβ_25__–__35_ oligomers and treated intraperitoneally once a day with vehicle, NX210c (2 mg/kg) from day 1 (D1) to D120 or with NX210c (2 mg/kg) from D11 to D38. Spontaneous alternation in the Y-maze was assessed weekly from D8 to D120 to study spatial working memory. Early and late-stage daily treatment with NX210c restored working memory deficit, as shown by the increased number of spontaneous alternations in the Y-maze compared with Aβ_25__–__35_-injected mice treated with vehicle. The data are presented as means and SEM. Two-way ANOVA followed by Tukey’s multiple comparisons test: ***p < 0.001, *p < 0.05 compared with Aβ_25__–__35_, *n* = 5 Ctrl, Aβ_25__–__35_, *n* = 6 Aβ_25__–__35_ + NX210c (D1–D120), Aβ_25__–__35_ + NX210c (D11–D38). **(B)** Mice were injected intracerebroventricularly with scrambled control peptides (Ctrl) or Aβ_25__–__35_ oligomers, and treated intraperitoneally once a day with vehicle, NX210c (0.1 mg/kg) along with donepezil (DPZ, 0.25 mg/kg) administered orally from D1 to D120, or successively with DPZ (1 mg/kg, D1–D43) and NX210c (2 mg/kg, D44–D78; 4 mg/kg, D79–D99; 8 mg/kg, D100–D113). Spontaneous alternation in the Y-maze was assessed weekly from D8 to D120 to study spatial working memory. Early stage daily treatment with a combination of sub-therapeutic doses of donepezil and NX210c restored working memory deficit, as shown by the increased number of spontaneous alternations in the Y-maze compared with Aβ_25__–__35_-injected mice treated with vehicle. Late-stage daily treatment with NX210c rescued donepezil loss of efficacy on working memory deficit. The data are presented as means and SEM. Two-way ANOVA followed by Tukey’s multiple comparisons test: ***p < 0.001, **p < 0.01, *p < 0.05 compared with Aβ_25__–__35_, *n* = 5 Ctrl, Aβ_25__–__35_, *n* = 6 Aβ_25__–__35_ + (NX210c + DPZ) (D1–D120), Aβ_25__–__35_ + DPZ + NX210c.

First, we sought to determine if the preventive effect of NX210c on long-term cognitive decline was sustained over time. Therefore, mice were treated with NX210c at 2 mg/kg, (i) once a day starting 1 h after administration of Aβ_25__–__35_ oligomers or (ii) once a day from 11 to 38 days post-injection of Aβ_25__–__35_ oligomers to mimic the clinical situation, by evaluating whether a transient 4-week daily treatment with NX210c when the pathology was already established (D11, Figure 2A), was still beneficial months after treatment arrest. Spatial working memory was evaluated weekly from D8 to D120 after injection of Aβ_25__–__35_ oligomers using the Y-Maze test (Figure 5A).

Long-term deficits in spatial working memory were sustained for up to 120 days after administration of Aβ_25__–__35_ oligomers [Figure 5A: *F*_48_,_288_ (mouse group × time) = 4.244, *p* < 0.0001. 77.2 and 45.9% of alternations in control and Aβ_25__–__35_-injected mice, respectively; *p* < 0.0001]. Early stage daily treatment with NX210c led to sustained recovery of working memory in Aβ_25__–__35_-injected mice (Figure 5A: 76.3% of alternations in Aβ_25__–__35_-injected mice treated with NX210c at D120; *p* < 0.0001 and >0.05 compared with Aβ_25__–__35_-injected mice treated with vehicle and control mice, respectively). Eight days after administration of Aβ_25__–__35_ oligomers, memory deficits were similar between Aβ_25__–__35_-injected mice treated with vehicle and Aβ_25__–__35_-injected mice that had not yet been started on NX210c treatment (Figure 5A: 73.9, 36.7, and 45.7% of alternations in control, and Aβ_25__–__35_-injected mice treated with vehicle and NX210c, respectively; *p* < 0.0001 compared with control, *p* > 0.05 between Aβ_25__–__35_-injected mice treated with vehicle and NX210c). Interestingly, 1 week later, Aβ_25__–__35_-injected mice treated with NX210c for only 4 days presented an increased number of alternations compared with that seen with Aβ_25__–__35_-injected mice treated with vehicle (Figure 5A: 77.9, 46, and 56.5% of alternations in control, and Aβ_25__–__35_-injected mice treated with vehicle and NX210c, respectively; *p* < 0.0001 compared with control, *p* = 0.0206 between Aβ_25__–__35_-injected mice treated with vehicle and NX210c). At 29 days after administration of Aβ_25__–__35_ oligomers, NX210c fully protected Aβ_25__–__35_-injected mice from spontaneous alternation impairments (Figure 5A: 77.4, 36.3, and 76.4% of alternations in control, and Aβ_25__–__35_-injected mice treated with vehicle and NX210c, respectively; *p* < 0.0001 and >0.05 compared with Aβ_25__–__35_-injected mice treated with vehicle and control mice, respectively). Despite the end of treatment at D38, the beneficial effect of NX210c in restoring working memory was sustained up to 4 months post-injection of Aβ_25__–__35_ oligomers (Figure 5A: 77.2, 45.9, and 76.6% of alternations in control, and Aβ_25__–__35_-injected mice treated with vehicle and NX210c, respectively; *p* < 0.0001 and >0.05 compared with Aβ_25__–__35_-injected mice treated with vehicle and control mice, respectively).

#### Early Stage Daily Combinatory Treatment With Donepezil/NX210c at Sub-Therapeutic Doses Prevents Long-Term Cognitive Decline in Aβ_25__–__35_-Injected Mice

In the same experiment, we also aimed to determine whether memory recovery was (i) sustained over time by using a combination of donepezil/NX210c at sub-therapeutic doses or (ii) rescued by NX210c as a second-line treatment following the loss of efficacy of donepezil. Therefore, mice were treated as follows: (i) daily combinatory treatment of donepezil and NX210c at sub-therapeutic doses (0.25 and 0.1 mg/kg doses, respectively) starting 1 h after administration of Aβ_25__–__35_ oligomers, or (ii) daily treatment with donepezil (1 mg/kg, active dose) until its sustained loss of efficacy was established (i.e., D43), followed by daily increasing doses of NX210c: 2 (D44–D78), 4 (D79–D99) and 8 mg/kg (D100–D113). Spatial working memory was evaluated weekly from D8 to D120 after injection of Aβ_25__–__35_ oligomers using the Y-Maze test (Figure 5B).

Interestingly, donepezil/NX210c at sub-therapeutic doses led to a sustained recovery of working memory in Aβ_25__–__35_-injected mice up to 4 months post-injection of Aβ_25__–__35_ oligomers [Figure 5B: *F*_48_,_288_ (mouse group × time) = 6.134, *p* < 0.0001. 77.2, 45.9, and 79.5% of alternations in control, and Aβ_25__–__35_-injected mice treated with vehicle and donepezil/NX210c, respectively; *p* < 0.0001 and >0.05 compared with Aβ_25__–__35_-injected mice treated with vehicle and control mice, respectively].

#### Second-Line Daily Treatment With NX210c Rescues Donepezil Loss of Efficacy on Long-Term Cognitive Decline in Aβ_25__–__35_-Injected Mice

Other Aβ_25__–__35_-injected mice treated with an active dose of donepezil were transiently protected from memory decline until D22 (Figure 5B: 72.1 and 79% of alternations in Aβ_25__–__35_-injected mice treated with donepezil at D8 and D22, respectively; *p* < 0.0001 and >0.05 compared with Aβ_25__–__35_-injected mice treated with vehicle and control mice, respectively). However, 1 week later, the efficacy of donepezil was significantly reduced (Figure 5B: 62.1% of alternations in Aβ_25__–__35_-injected mice treated with donepezil; *p* < 0.0001 and >0.001 compared with Aβ_25__–__35_-injected mice treated with vehicle and control mice, respectively) and was found to have ceased for the following 2 weeks (Figure 5B: 36.2 and 38.7% of alternations in Aβ_25__–__35_-injected mice treated with donepezil at D29 and D36, respectively; *p* > 0.05 and <0.0001 compared with Aβ_25__–__35_-injected mice treated with vehicle and control mice, respectively). Aβ_25__–__35_-injected mice treated with donepezil were treated with NX210c (2 mg/kg) from D44 to determine the efficacy of NX210c as a second-line treatment. Interestingly, NX210c progressively rescued the decrease in the number of alternations induced by the loss of efficacy of donepezil (Figure 5B: 55.1 and 67.9% of alternations in Aβ_25__–__35_-injected mice treated with donepezil + NX210c at D50 and D57, respectively; *p* = 0.0014 and <0.0001 compared with Aβ_25__–__35_-injected mice treated with vehicle, and *p* < 0.0001 and >0.05 compared with control mice). However, rescue of spatial working memory was only temporary and increasing the dose of NX210c to 4 mg/kg from D79 until D99 did not result in reversion back to the basal number of alternations (Figure 5B: 56.8 and 60.5% of alternations in Aβ_25__–__35_-injected mice treated with donepezil + NX210c at D85 and D99, respectively; *p* = 0.0387 and <0.0001 compared with Aβ_25__–__35_-injected mice treated with vehicle, and *p* < 0.0001 and >0.0002 compared with control mice). Finally, mice were treated with a higher dose of NX210c (8 mg/kg) from D100 which restored the number of alternations to the basal levels seen in control mice until D120 despite the end of the treatment at D113 (Figure 5B: 68.9 and 79.8% of alternations in Aβ_25__–__35_-injected mice treated with donepezil + NX210c at D106 and D120, respectively; *p* < 0.0001 and >0.05 compared with Aβ_25__–__35_-injected mice treated with vehicle and control mice, respectively).

## Discussion

To our knowledge, this is the first study to show that the SCO-spondin-derived peptide NX210 and its cyclic form, NX210c, represent potent treatment agents for reversing learning and memory deficits in the Aβ_25__–__35_-induced mouse model of AD. Indeed, systemic administration of NX210 and NX210c restored learning and short- and long-term memory regardless of the therapy paradigm used (early or late stand-alone treatments, combination with donepezil or second-line treatment). Furthermore, the therapeutic effect of these drugs was maintained over time for up to 4 months and was accompanied by sub-chronic decreased levels of pathological hallmarks of AD, such as Aβ_1__–__42_ and pTau, and increased synaptogenesis.

Peptides are highly selective, efficient, and well-tolerated drugs, with better safety profiles compared to those of small molecules, antibodies and biologics (Fosgerau and Hoffmann, 2015). Over 70 peptide drugs are marketed worldwide and more than 150 are in different phases of clinical trials (Lau and Dunn, 2018; Lee and Hur, 2019; King and Suphioglu, 2020). Indeed, the development of peptide therapeutics includes peptide inhibitors of Aβ and β-site amyloid precursor protein (APP) cleaving enzyme 1 for the treatment of AD (Baig et al., 2018; King and Suphioglu, 2020). NX210 is a small peptide designed from the most conserved sequence of the TSR1 motif of SCO-spondin and has two cysteines that create a disulfide bond under oxidative conditions, resulting in an alternative cyclic form of NX210, designated NX210c. To date, the therapeutic effect of NX210 has only been studied in the context of spinal cord injury, where it was administered locally to promote neurorepair in rats (Sakka et al., 2014). Here, we describe for the first time that repeated systemic injections of the native or oxidized cyclic forms of this peptide efficiently reduced the levels of common hallmarks of AD pathology and restored learning and memory at both early and late disease stages. The prevention of cognitive deficits with NX210c is observed at a lower dose than that with NX210, which can be explained by our internal data showing the fast cyclization of NX210 following systemic administration, suggesting that NX210c may represent the active form of the peptide. Liquid chromatography tandem mass spectrometry studies are warranted to determine whether intraperitoneal injections of NX210 lead to a full conversion of NX210 into NX210c or whether remnants of NX210 are present. Currently, it is unclear whether NX210 and/or NX210c cross the blood-brain barrier (BBB); however, fixation of Aβ_25__–__35_ or Aβ_1__–__42_ to the receptor for advanced glycation end products decreases the viability of endothelial cells and alters tight junction protein levels, thereby compromising BBB integrity *in vitro* and *in vivo* (Kook et al., 2012; Chumakov et al., 2015; Wan et al., 2015; Miranda-Azpiazu et al., 2018; Cuevas et al., 2019). This, in turn, may help NX peptides reach the brain in the Aβ_25__–__35_ mouse model of AD used in this study.

The mechanisms of action of NX210 or NX210c remain to be explored in more detail; however, β_1_-integrin may represent a putative target of NX peptides to mediate neuroprotection against Aβ_25__–__35_ oligomers (Caltagarone et al., 2007) and the subsequent effects on cognitive function in Aβ_25__–__35_-injected mice treated with NX210 and NX210c. This hypothesis is supported by the protective effects of NX210 and NX210c against apoptosis, necrosis, and neurite growth retraction observed in rat and human primary cortical and hippocampal neuron cultures subjected to glutamate-induced excitotoxicity (Delétage et al., 2021), a common feature of AD (Dong et al., 2009). NX210 and NX210c disrupt the apoptotic machinery by reducing cytochrome c release and caspase activation, and promote the activation of the phosphoinositide 3-kinase/mammalian target of rapamycin (PI3K/mTor) pro-survival pathway (Delétage et al., 2021). These effects are in line with reduction of caspase 12 levels and increase in the levels of synaptic markers synaptophysin and PSD95 observed in the prefrontal cortex of mice administered with Aβ_25__–__35_ oligomers and treated with NX210 and NX210c. Interestingly, the neuroprotective effects of NX210 and NX210c were terminated in the presence of an antibody anti-β_1_-integrin (Delétage et al., 2021). These results are in line with that of a previous study showing that NX210 increases neurite growth and root count in the B104 neuronal cell line via β_1_-integrin (Bamdad et al., 2004). Similarly, integrin-specific binding of short synthetic peptides promotes neuronal survival and neurite growth in rat primary cortical neuron cultures (Kuwar et al., 2020). Furthermore, extracellular matrix proteins such as laminin, fibronectin, and type 1 collagen may protect the SK-N-BE and SH-SY5Y neuronal cell lines from Aβ_25__–__35_-induced apoptosis by preventing Aβ_25__–__35_ binding to β_1_-integrin (Bozzo et al., 2004; Caltagarone et al., 2007). Similarly, pre-treatment of primary cultures of rat hippocampal neurons with selective antagonists of αv or β_1_ integrin subunits prior to Aβ_1__–__42_ exposure reduces Aβ_1__–__42_-dependent increase in levels and phosphorylation of the integrin downstream effector, focal adhesion kinase (FAK), which reduces the number of apoptotic cells and increases neuronal survival (Han et al., 2013). In addition, Woo et al. (2015) demonstrated that Aβ_1__–__42_ oligomers bind to β_1_-integrin with a high affinity, which leads to the misconformation and subsequent loss of surface β_1_-integrin, thereby triggering apoptosis and decreasing the levels of synaptic markers via the mitochondrial translocation/activation of cofilin using various culture models. Heterozygous knockout of cofilin fully restores synaptic marker levels and long-term potentiation in the hippocampus, which alleviates contextual memory impairments in APP/presenilin-1 (PS1) transgenic mice (Woo et al., 2015). A specific RGDS peptide (which prevents cell-extracellular matrix adhesion) and an anti-cluster of differentiation 29 (CD29) antibody targeting β_1_-integrin also prevent Aβ oligomer-induced increase of β_1_-integrin levels and nicotinamide adenine dinucleotide phosphate oxidase isoform 2 (NOX2)-dependent overexpression of GFAP in primary cultures of cerebral cortical astrocytes (Wyssenbach et al., 2016). Furthermore, the anti-CD29 antibody prevents the increase in NOX2 levels and subsequent astrogliosis induced by intrahippocampal injection of Aβ oligomers in mice (Wyssenbach et al., 2016). This is in line with increased protein levels of β_1_-integrin, NOX2 and GFAP in hippocampal astrocytes of 18-month-old triple-transgenic (3 × Tg)-AD mice with mutations in APP, PS1 and tau and in postmortem prefrontal cortices of patients with AD (Wyssenbach et al., 2016). In this study, we demonstrated that the treatment of Aβ_25__–__35_-injected mice with NX210 or NX210c restored the protein levels of GFAP and synaptic markers in the prefrontal cortex 2 weeks after injection of Aβ_25__–__35_. Taken together, these studies suggest that the putative binding of NX210 or NX210c to β_1_-integrin may prevent β_1_-integrin-dependent neurotoxicity and astrogliosis triggered by Aβ_25__–__35_ and Aβ_1__–__42_ oligomers in the Aβ_25__–__35_ mouse model of AD. However, it remains to be determined whether increased cerebral levels of Aβ_1__–__42_ induced by Aβ_25__–__35_ oligomers result in the formation of Aβ_1__–__42_ oligomers and whether the beneficial effect of delayed treatment with NX210 and NX210c on cognitive impairment is due to disruption of the fixation of Aβ_1__–__42_ oligomers to β_1_-integrin. In order to confirm the involvement of β_1_-integrin in the therapeutic effects of NX210 and NX210c in AD, we would need to determine the following: (i) whether levels of β_1_-integrin are differentially modulated in neurons (decrease) and astrocytes (increase) in the Aβ_25__–__35_ mouse model used in this study and (ii) whether NX210 and NX210c prevent cofilin activation and decrease the levels of FAK (in neurons) and NOX2 (in astrocytes) in the prefrontal cortex and hippocampus of Aβ_25__–__35_-injected mice.

Disturbances in the cholinergic and glutamatergic systems are common features of AD that contribute to learning and memory impairments (Ferreira-Vieira et al., 2016; Calvo-Flores Guzmán et al., 2018; Conway, 2020). Therapies that address the decrease in acetylcholine levels or the hyperactivity of NMDA receptors represent the current standards of care for the treatment of AD. These include use of acetylcholinesterase inhibitors donepezil, galantamine and rivastigmine, and the uncompetitive NMDA receptor antagonist memantine (Zhang et al., 2020). However, none of these drugs slow or halt the progression of neural damage and subsequent loss of neurons, which are responsible for the symptoms and fatal outcome of AD. Furthermore, their effectiveness in the symptomatic control of AD varies from person to person and is limited in duration. Although donepezil is one of the mainstay treatments for patients with mild to severe AD, its clinical efficacy in improving cognitive decline, including memory loss, is rather modest, and targeting the cholinergic system results in adverse dose-dependent effects on peripheral organs, such as gastrointestinal symptoms and urinary infections, which may lead patients to prematurely discontinue their treatment (Tsoi et al., 2016; Arvanitakis et al., 2019; Haake et al., 2020). In view of improving treatment efficacy for cognitive disorders, combinatory treatments, such as combination of donepezil and memantine (Namzaric^®^), which was approved by the Food and Drug Administration in 2014 for patients with moderate to severe AD who were stabilized with donepezil (Cummings et al., 2019; Guo et al., 2020), have gained much attention in the past few years. However, this combination (Acrescent) was not approved by the European Medicines Agency, which considered that the risk/benefit ratio for the fixed combination was not favorable. Indeed, the uncertainties regarding the efficacy of this combinatory approach on memory, along with cumulative risk of adverse effects, reveal the urgent need to find disease-modifying therapies as stand-alone treatment or combinatory treatments with current first-line therapies (Yatabe et al., 2013; Cummings et al., 2019).

Preclinical studies suggest that the therapeutic benefits of donepezil on memory loss and cognitive decline may not rely solely on acetylcholine-related symptomatic effects but may also involve neuroprotective mechanisms (Kim et al., 2017). Donepezil protects rat primary cortical neuron cultures from oxygen-glucose deprivation and NMDA-induced excitotoxicity, and rat primary septal neuron cultures from Aβ toxicity, in a dose-dependent manner (Akasofu et al., 2008). Donepezil (0.5 mg/kg) also partially protects AD-like mice from LPO in the Aβ_25__–__35_ model (Meunier et al., 2006). This neuroprotective action of donepezil is in line with our results that showed a decrease in Aβ_1__–__42_, pTau and TNF-α levels, astrogliosis, LPO and endoplasmic reticulum stress, along with an increase in the levels of synaptic markers synaptophysin and PSD95, in Aβ_25__–__35_-injected mice treated with donepezil at 1 mg/kg for 11 days. The association of sub-therapeutic doses of donepezil (0.25 mg/kg; intraperitoneal route) with PRE-084 or ANAVEX2-73 (selective and non-selective agonists of Sigma1 receptor, respectively; 0.1 mg/kg) administered 20 min prior to Aβ_25__–__35_ oligomers prevents the establishment of spatial working memory and contextual long-term memory deficits in mice (Maurice, 2016). Droguerre et al. (2020) also used the same model and showed that the combination of sub-therapeutic doses of donepezil (0.25 mg/kg; oral route) and mefloquine (0.3 and 1 mg/kg) administered daily starting 1 day post-injection of Aβ_25__–__35_ oligomers restored recognition memory and spatial long-term learning and/or memory deficits. Similarly, we showed that daily treatment with sub-therapeutic doses of donepezil (0.25 mg/kg; oral route) along with sub-therapeutic doses of NX210 or NX210c (0.1 mg/kg) prevented spatial working and contextual long-term memory decline 8–10 days post-injection of Aβ_25__–__35_ oligomers, respectively. This therapeutic effect of NX210c added to donepezil on spatial working memory was maintained at a 4-month follow-up. Although donepezil (0.1 or 1 mg/kg; oral route) increases the number of spontaneous alternations during the Y-maze test performed by 50-week-old APP/PS1 mice, it progressively loses its efficacy after 3 weeks of treatment (Kim et al., 2016). In this study, we observed a loss of efficacy of donepezil (1 mg/kg; oral route) after 4 weeks of treatment; 1 week later, donepezil was no longer effective. Interestingly, NX210c at 2 mg/kg completely reverted donepezil resistance; however, the concentrations needed to be increased up to 8 mg/kg to maintain restored spatial working memory, which is likely due to the fact that the peptide was administered for the first time at a significantly late stage of disease progression (i.e., D44).

In addition to NX210 and NX210c, several stand-alone therapies have also been shown to be effective in the Aβ_25__–__35_ mouse model. For example, enantiomer neurosteroids administered intracerebroventricularly at the same time as Aβ_25__–__35_ oligomer injection also have beneficial effects on spatial working memory and contextual long-term memory in a dose-dependent manner (El Bitar et al., 2014). Similarly, subcutaneous injections of a fusion protein targeting TNF-α when administered every other day reduce spatial working memory and contextual long-term deficits in the Aβ_25__–__35_ mouse model (Detrait et al., 2014). A combination of photobiomodulation with a static magnetic field using a RGn500 device applied simultaneously on the head and abdomen once a day for 20 min decreased Aβ_1__–__42_, pTau and TNF-α protein levels, LPO and glial activation in Aβ_25__–__35_-injected mice (Blivet et al., 2018). Ultimately, spatial working memory and contextual long-term memory were improved in treated Aβ_25__–__35_-injected mice (Blivet et al., 2018). However, none of these studies addressed the long-term effect of their therapeutic approaches on memory deficits beyond 16 days after injection of Aβ_25__–__35_ oligomers (Maurice et al., 1996; Detrait et al., 2014; El Bitar et al., 2014; Blivet et al., 2018; Droguerre et al., 2020). Meanwhile, we observed a sustained effect of our treatment with the peptide for up to 4 months after Aβ_25__–__35_ injection in various experimental paradigms (early or late stand-alone treatments, combination with donepezil or second-line treatment). Furthermore, we reported the normalization of many cerebral pathological markers and features, including Aβ_1__–__42_, pTau, TNF-α, synaptophysin, PSD95, LPO and astrogliosis, in Aβ_25__–__35_-injected mice treated with NX210 and NX210c. In addition, NX210 and NX210c were administered via the systemic route, which facilitates their use in clinical conditions.

The Aβ_25__–__35_ mouse model only relies on the amyloid hypothesis wherein Aβ triggers AD pathology due to accumulation and oligomerization, which in turn trigger tau phosphorylation, neuroinflammation, oxidative stress and synaptic dysfunction, common features described in patients with AD (Heneka et al., 2015; Selkoe and Hardy, 2016; Jackson et al., 2019; Ayton and Bush, 2021). This mouse model is very convenient to establish due to the rapid development of AD pathology and subsequent induction of learning and memory deficits within 1–2 weeks, which allows rapid screening of new potential therapeutics. However, this model does not display amyloid plaques and neurofibrillary tangles, which are two major hallmarks of AD. Furthermore, despite several studies reporting death of hippocampal pyramidal neurons in the Aβ_25__–__35_ mouse model (Villard et al., 2009; Maurice et al., 2013; Chumakov et al., 2015; Reggiani et al., 2016), no consensus on neuronal death in this model was found in our experiments (data not shown). Thus, further studies are warranted to assess the effect of NX210c on amyloid plaque deposition and neuronal death by using other AD models such as the APP/PS1 mouse model (Jankowsky et al., 2004), the 3 × Tg-AD mouse model with mutations in APP, PS1, and tau genes (Oddo et al., 2003), and the 5 × familial AD mouse model with five mutations in APP and PS1 genes (Oakley et al., 2006). It would also be worthwhile to examine the preventive effect of NX210c on early vascular dysfunctions, such as BBB breakdown, pericyte loss or perfusion deficits, which can initiate amyloid and tau pathogenic cascades (Montagne et al., 2015, 2017; Nation et al., 2019). Indeed, one hypothesis is that NX210 and NX210c may have prevented Aβ_25__–__35_ oligomer-induced BBB dysfunction, as described previously *in vitro* and *in vivo* in the mouse model used in this study (Kook et al., 2012; Chumakov et al., 2015; Wan et al., 2015; Miranda-Azpiazu et al., 2018; Cuevas et al., 2019). This may have led to a halt in the progression of AD and cognitive deficits. In order to confirm this hypothesis, we would need to look at the extravasation of Evans blue or fibrinogen from the blood to the brain in mice administered with scrambled control peptides and Aβ_25__–__35_ oligomers and treated or not with NX210 and NX210c. In AD progression, synaptic impairment in the cortex and the hippocampus precedes neuronal death and is associated with cognitive impairments (Yuki et al., 2014; Wang et al., 2016; Jackson et al., 2019). As an alternative or in addition to a putative role of NX peptides on neuronal death in the Aβ_25__–__35_ mouse model, we have also shown that NX210 and NX210c restored Aβ_25__–__35_-induced synaptic disruption by reducing levels of synaptophysin and PSD-95 which are pre-synaptic and post-synaptic markers, respectively. Synapse integrity was presumably improved in Aβ_25__–__35_-injected mice treated with NX210 and NX210c either (i) by decreasing Aβ_1__–__42_ levels in the prefrontal cortex and the hippocampus which in turn reduced Aβ_1__–__42_ oligomer-induced increase in synaptophysin and PSD-95 levels (Yuki et al., 2014; Wang et al., 2016), or (ii) by activating the PI3K/mTor pathway (Delétage et al., 2021) which in turn increased the levels of synaptic proteins.

## Conclusion

This study provides the first evidence that the SCO-spondin-derived peptide, NX210, especially its cyclic form (NX210c), reduces common hallmarks of AD pathology and restores learning and memory at both early and late disease stages. Interestingly, complete interruption of the treatment did not affect memory improvements over time, highlighting the possible disease-modifying effect of this peptide. As a second-line treatment, this peptide reduced the rate of memory deficits observed after loss of efficacy of donepezil. Finally, sub-therapeutic doses of the peptide and donepezil act synergistically to fully restore memory. In addition to having a slightly better efficacy/dose ratio than NX210 in this AD mouse model, NX210c stability and scale-up manufacturing are also more convenient than those of the linear form, which justifies to evaluate NX210c as a drug candidate in the next clinical steps rather than NX210.

Further studies at the preclinical stage are now warranted (i) to investigate whether the mechanism of action of NX210c in AD pathology involves preventing the fixation of Aβ peptides to β_1_-integrin, thereby contributing to restoring memory function; (ii) to confirm the therapeutic effects of NX210c in other AD models; and (iii) to investigate the therapeutic effects of NX210c in animal models of other neurological diseases associated with cognitive deficits. Overall, this study reveals the therapeutic potential of an innovative disease-modifying peptide to reduce AD hallmarks and, ultimately, restore learning and memory functions in patients with AD.

## Data Availability Statement

The datasets presented in this article are not readily available because the requester needs to be qualified by the authors beforehand. Requests to access the datasets should be directed to JL, jledouce@axoltis.com and SL, slemarchant@axoltis.com.

## Ethics Statement

The animal study was reviewed and approved by the French Ministry of Research and all procedures were conducted in strict adherence to the European Union Directive of September 22, 2010 (2010/63/UE).

## Author Contributions

JL, ND, VB, SL, and YG designed the research and analyzed and/or interpreted the data. JL and SL wrote the manuscript and prepared the figures. ND, VB, and YG provided critical comments on the draft of the manuscript. All the authors read and approved the final version of the manuscript.

## Conflict of Interest

JL, ND, VB, and SL are employed by Axoltis Pharma. VB is Chief Medical Officer of Axoltis Pharma. YG is President of Godfrin Life-Sciences giving scientific advices to Axoltis Pharma, and is also Chief Executive Officer and a shareholder of Axoltis Pharma.
